# Familial hemiplegic migraine type 2 due to a novel missense mutation in *ATP1A2*

**DOI:** 10.1186/s10194-021-01221-x

**Published:** 2021-03-12

**Authors:** Fabio Antonaci, Sabrina Ravaglia, Gaetano S. Grieco, Stella Gagliardi, Cristina Cereda, Alfredo Costa

**Affiliations:** 1 IRCCS Mondino Foundation, via Mondino 2, 27100 Pavia, Italy; 2 Department of Brain and Behavioral Sciences, University of Pavia, Pavia, Italy; 3 Genomic and Post-Genomic Unit, IRCCS Mondino Foundation, Pavia, Italy

**Keywords:** Familiar hemiplegic migraine, Migraine with Aura, *ATP1A2* gene

## Abstract

**Background:**

The mechanisms of genotype-phenotype interaction in Familiar Hemiplegic migraine type 2 (FHM2) are still far from clear. Different *ATP1A2* mutations have been described, with a spectrum of phenotypes ranging from mild to severe. No genotype-phenotype correlations have been attempted.

**Case presentation:**

We describe an Italian family with FHM and a missense *ATP1A2* variant (L425H) not previously described. The clinical picture was mild in all the affected members.

**Conclusions:**

Co-segregation of the variant with the aura phenotype was complete in this family, suggesting a 100% penetrance. In silico protein prediction softwares indicate that this variant may change the 3D structure of ATPA1A2 at the cytoplasmic loop between the two central transmembrane helices. Milder FHM phenotypes are rarely reported in literature, likely because case reports are biased towards the most severe phenotypes, with milder forms possibly misdiagnosed as sporadic migraine with aura forms (MAs), even with complex auras. Further studies taking into account intra-familiar variability and functional consequences on the channel protein may help clarify genotype-phenotype correlations.

## Background

Hemiplegic migraine (HM) is a rare form of migraine with aura (MA) in which attacks are characterized, among other symptoms, by complex auras including motor disturbances, often lasting several days. A subset of HMs, labelled as familiar HM type 2 (FHM2), are associated with mutations in the *ATP1A2* gene [1]. *ATP1A2* encodes for the alpha2-subunit (the catalytic site) of the Na/K ATPase, regulating the excitable properties of muscle and nerve cells [1]. All mutations are thought to be loss-of-function [1, 2], leading to decreased clearance, and thus to increased levels of glutamate in the synaptic cleft: this would facilitate cortical excitability and cortical spreading depression, leading to aura [2]. Different *ATP1A2* mutations have been described, with a spectrum of phenotypes ranging from mild to severe: these latter include permanent neurological signs and MRI abnormalities in the form of vascular lesions [3]. However, studies on genotype-phenotype correlations are still lacking [1, 4].

We here describe an Italian family with FHM and a missense *ATP1A2* variant not previously described. The clinical picture was mild in all the affected members. The pathogenicity of this new L425H variant was supported by a consistent clinical picture, in addition to a mutation predictive software.

## Methods

### Case report

A 21-year-old male was admitted to our Institute for a family and personal history of sporadic MA; associated episodes of migraine without aura (MWA) were also reported. Frequency for MWA was 2–3 episodes per month, whereas that of MA was of a total of three episodes over the previous ten years. The patient had reported a first episode of visual, followed by aphasic, followed by sensory aura, accompanied by headache, when he was 10; at that time no examinations were performed. Since then, two further episodes of MA had occurred: the aura presented in the form of visual symptoms, followed by aphasia (with the features of posterior aphasia), followed by hemi-paresthesia (starting from one hand and then spreading to the whole upper limb, and in only one occasion to the lower limb), with full recovery of neurological symptoms within 24 h. Psychological stress was reported as possible trigger. Only the third episode was associated with right hemiparesis, of mild degree (he was able to walk without assistance during aura) so that he was admitted to the emergency room and then hospitalized since an acute vascular accident was hypothesized. Neurological symptoms gradually disappeared a few hours after hospitalization. All instrumental examinations were performed on the day after admission, when both aura symptoms and headache had subsided. Supra-aortic vessel Doppler-sonography and transcranial Doppler-sonography were normal. EEG, performed on the day after admission after the aura had subsided, revealed slowing of activity, with delta waves, prominent on parieto-occipital areas and only involving the left hemisphere (contralateral to the neurological symptoms). Brain MRI, including angio-MRI and DTI, was normal. The neurological examination was normal outside the episodes of MWA. Due to the low frequency of the aura episodes, which were never associated with severe functional deficits, no prophylactic treatment was advised.

#### Family history

**Fig. 1:**
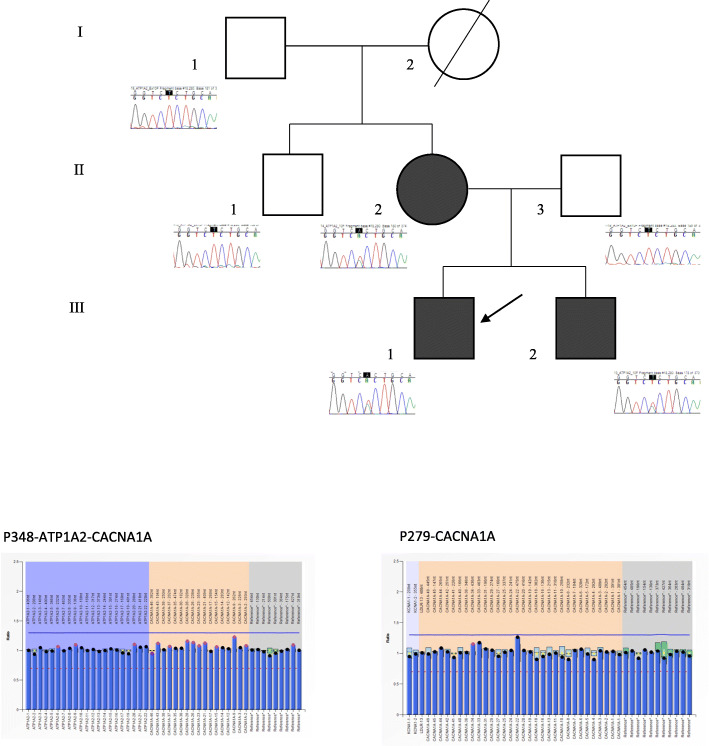
Pedigree of the family and Sanger sequencing results. Genetic analysis was performed in 6 consanguineous patients of three generations, and revealed a dominant inheritance leading to the missense mutation L425H. Patient I-2 is deceased; she had complained of “limb paresthesias” followed by headache in her childhood. All the affected members were symptomatic for sporadic migraine with aura (II-2, III-1 and -2); II-2 and III-2 also had sporadic episodes of migraine without aura. The proband (III-2) was the only member with an episode of hemiplegic migraine, while the others only showed complex auras (visual, aphasic, sensory)

Data from six consanguineous individuals were collected (Fig. 1). All provided informed consent for genetic analysis. The 23-year-old brother of the proband reported a first episode of MA at 16, characterized by visual aura followed by hemi-paresthesia, dysgraphia and aphasia; the full aura symptoms resolved within one hour. Brain MRI and angio-MRI were normal. Since then he had been experiencing further episodes of aura, with similar features, with a frequency of about one episode per year.

The 54-year-old proband’s mother reported episodes of migraine with visual and sensory aura since age 23, together with sporadic episodes of MWA, with a frequency of less than one episode per year, and a tendency to improve in frequency in more recent years (she had had no episodes over the last 4 years). Her mother, not available for genetic analysis, had reported transient episodes of “limb paresthesias” followed by headache in her young adulthood. The 59-year-old father did not report episodes of headache and his genetic screening was negative.

#### Follow-up

Since diagnosis, the patient, his brother and his mother have been followed – up yearly for at least five years; the disease course and clinical manifestations still remain unchanged in the proband and his brother, while the mother has not experienced further episodes since then and thus over the last 9 years None of the three affected members needed prophylactic migraine treatment due to the low frequency of both aura and headache episodes; attacks were treated with NSAIDs.

### DNA preparation and genetic analysis

Genomic DNA (gDNA) was isolated from peripheral blood leukocytes with an automated purification system Maxwell 16 (Promega). NGS analysis was performed using the MiSeq TruSeq Custom Amplicon protocol for *CACNA1A, ATP1A2, SCN1A, PRRT2* (Illumina) according to the manufacturer’s instructions. Sequencing was done using the 151 bp paired-end v3 chemistry on the Illumina NextSeq 550 platform (Illumina). The Illumina fastq sequencing data were mapped to the human reference assembly, hg19 (GRCh37; UCSC genome browser) and the downstream bioinformatic processing was done as reported elsewhere [5]. Produced VCFs were processed with eVAI software (enGenome; https://evai.engenome.com/#login) for annotation. Samples were also investigated for deletions or duplications using SALSA MLPA probemix P348-*ATP1A2*-*CACNA1A* and P279-*CACNA1A* assay (MRC-Holland). The assay was performed according to the manufacturer’s recommendation and data analyzed using Coffalyser. Net software.

### Variant analysis

Variant were retained according to the following criteria: i) variants with minor allele frequency (MAF) < 0.01 on the ExAC, GnomAD, dbSNP and ESP6500 databases; ii) variants that fall in the gene coding regions, splice-site regions and variants that disrupt the 3′/5′-untranslated regions (UTRs); iii) variants with a read depth > 15. Retained variants and relative genes were further screened after their investigation on different databases, such as OMIM (Online Mendelian Inheritance in Man; https://www.omim.org/), Decipher V9.29 (https://decipher.sanger.ac.uk/), HSF (Human Splicing Finder; http://www.umd.be/HSF/), MGI (Mouse Genome Informatics; http://www.informatics.jax.org/), STRING (https://string-db.org/) and GTEx (Genotype-Tissue Expression; https://gtexportal.org/home/). Variants retained were further investigated using different software and databases: VarSome software (https://varsome.com/), to classify variants according to the guidelines of the American College of Medical Genetics (ACMG criteria) [6]; dbSNP and ClinVar, if variants have been already described and associated with specific diseases; in silico prediction software (SIFT, Polyphen-2, Mutation Taster, etc) to evaluate the physical-chemical consequence of an amino acid change on protein structure and functionality. Final retained variants were validated using conventional Sanger sequencing.

### Prediction of structure of the mutant protein

The exact 3D-structure of ATP1A2 is unknown. However, we used the software HOPE (https://www3.cmbi.umcn.nl/hope) to build a model of this protein, based on a homologous structure. The model is built using the Yasara & WHAT IF Twinset. Structural information is collected using information from WHAT IF Web services, the UniProt database and the Reprof software.

## Results

Analysis of *ATP1A2* gene revealed in the proband the previously unknown variant c.1274 T > A in heterozygosity (*ATP1A2*:NM_000702.4:exon10:c.1274 T > A:p.L425H), leading to a histidine substitution of a leucine in position 425 (p.L425H) in exon 10.

All prediction softwares used (SIFT, Polyphen-2, and Mutation Taster), defined this variant as deleterious and damaging. The founded variant ATP1A2:L425H, based on the ACMG classification [6], is defined as “Uncertain Significance”. The calculated criteria are: PM2 moderate, PP2 and PP3 supporting, according to VarSome (https://varsome.com/variant/hg19/ATP1A2%3AL425H) [7]. In details: this variant was not found in gnomAD exomes (PM2 moderate); 49 out of 52 non-VUS missense variants in gene *ATP1A2* are pathogenic (PP2 supporting); pathogenic computational verdict based on 11 pathogenic predictions and one benign prediction only, support a deleterious effect of this variant on the gene or gene product (PP3). Based on these considerations, this variant may be defined as “pathogenic”.

With regard to structural prediction, the wild-type residue is predicted (using the Reprof software) to be located in an α-helix. In particular, this area corresponds to the cytoplasmic loop between transmembrane helices 4 and 5 (TM 4 and TM5), containing the phosphorylation and nucleotide binding domains.

The sequence included in the loop between TM4 and TM5 is highly conserved through species [8]. The same variant was also found in the brother and the mother of the proband, while it was absent in the asymptomatic relatives (Fig. 1). Thus, the transmission appears dominant with a complete penetrance, since all the affected members were symptomatic. The clinical picture was so mild that some symptoms could easily be under-recognized: indeed, before hospitalization of the proband, the mother had never searched for medical attention for her symptoms; rather, she felt safe about her aura symptoms since her mother also had complained of a similar clinical picture of transient sensory symptoms followed by headache since her young adulthood, with a tendency to disappear in subsequent years.

**Fig. 2:**
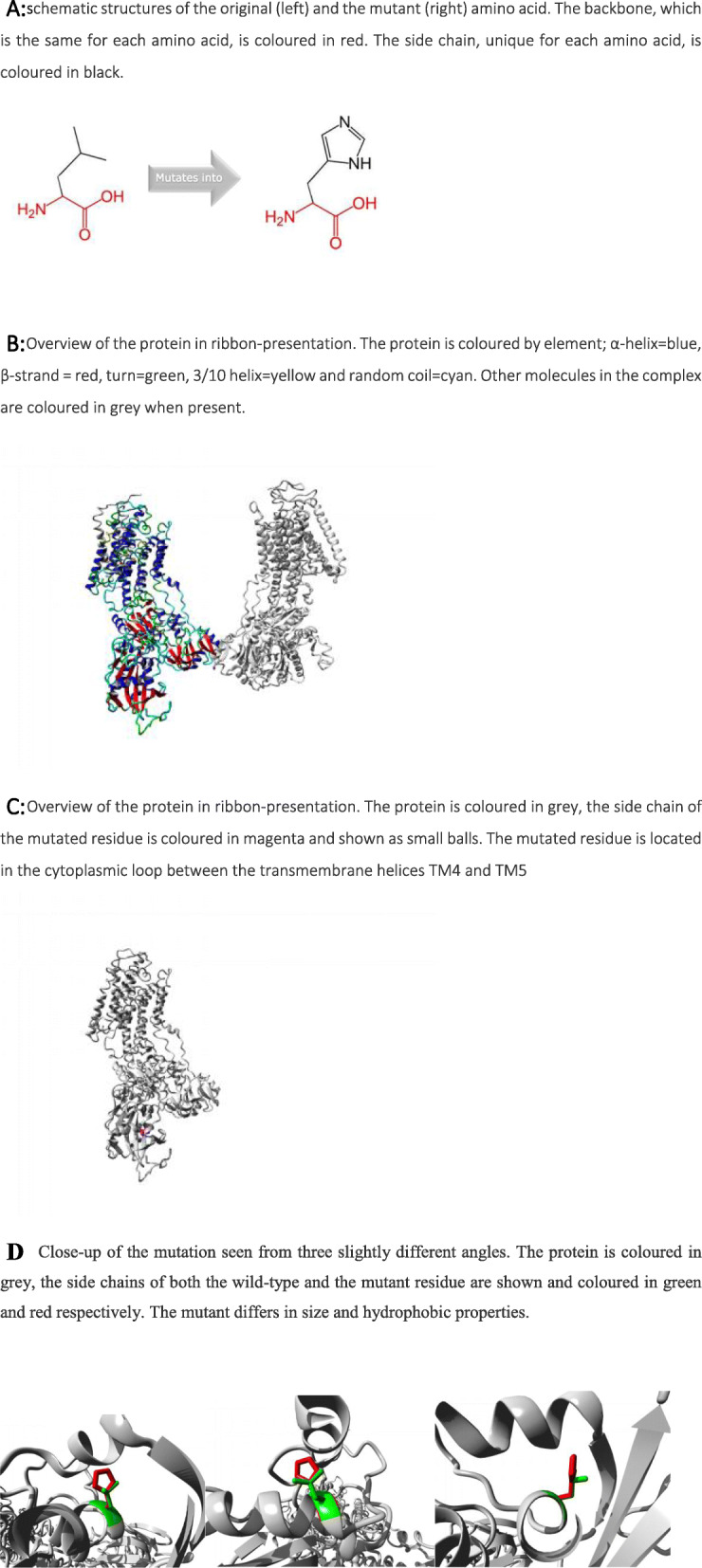
Evaluation of the secondary structure prediction of the L425H variant by molecular modelling. **2A:** schematic structures of the original (left) and the mutant (right) amino acid. The backbone, which is the same for each amino acid, is coloured in red. The side chain, unique for each amino acid, is coloured in black. **2B:** Overview of the protein in ribbon-presentation. The protein is coloured by element; α-helix = blue, β-strand = red, turn = green, 3/10 helix = yellow and random coil = cyan. Other molecules in the complex are coloured in grey when present. **2C:** Overview of the protein in ribbon-presentation. The protein is coloured in grey, the side chain of the mutated residue is coloured in magenta and shown as small balls. The mutated residue is located in the cytoplasmic loop between the transmembrane helices TM4 and TM5. **2D** Close-up of the mutation seen from three slightly different angles. The protein is coloured in grey, the side chains of both the wild-type and the mutant residue are shown and coloured in green and red respectively. The mutant differs in size and hydrophobic properties

Prediction of pathogenicity through dedicated software is illustrated in Fig. 2A-D. Structural analysis of the L425H variant using a prediction model [9] shows that this variant may affect the structure and function of the protein. The mutant residue is bigger and more hydrophobic than the wild-type, and appears likely to cause a change in interactions and structure. This variant is expected to convert the wild-type residue in a residue that does not prefer α-helices as secondary structure.

## Discussion

In all members of our family with FHM2 the aura was visual, aphasic (in the form of posterior aphasia), and sensory; only one member (the proband) experienced a single episode of transient hemiparesis, that was actually the reason for his hospitalization, followed by investigation on his family history and thus to genetic analysis of all members. All aura episodes were lasting less than 24 h and occurred at a very low frequency, so that the overall disease course was benign in all the affected members. In two of the three affected members MA co-existed with MWA. Neurological examination and brain MRI were normal in all subjects, there were no comorbidities other than headache. The time-and-space pattern of the reported auras followed a cortical distribution (visual, then aphasic, then sensory, and eventually motor in the proband only and in one episode only), consistent with the spreading depression mechanism.

Mutations of *ATP1A2* gene have been described in familiar hemiplegic migraine as dominant mutations, usually missense, which are thought to cause loss-of-function [1]. At least 80 different mutations have been described (https://databases.lovd.nl/shared/genes/ATP1A2), in most cases without ascertainment of their pathogenic role, except for the association with the aura phenotype. No genotype-phenotype correlations have been attempted [3].

In this family we found a novel *ATP1A2* variant, that we believe to be pathogenetic since it was present in all three affected member of the family, while it was absent in the three healthy relatives. The inheritance pattern is fully consistent with an autosomal dominant condition, although with a very mild clinical expressivity: this family shows a particularly benign disease course, rarely reported in literature. The disease spectrum, as described in literature, ranges from mild to severe, with several patients affected by additional and persistent neurological features such as intellectual disability, deafness, epilepsy, and personality disturbances [3, 10]; indeed, most patients require prophylactic headache treatment due to severity, duration (even several weeks) and frequency of their MA episodes [3, 10]. Severe MRI abnormalities lasting several weeks and including marked hemispheric edema with mass effect are described [3]. However, literature case reports are likely to be biased towards the most severe phenotypes, with milder forms being possibly misdiagnosed as sporadic MAs, even with complex auras.

Beyond the association with the classical clinical picture of MA, other arguments suggesting the pathogenicity of this variant derive from the analysis of the predicted results on protein structure. Prediction of the functional effect was assessed by a dedicated software [9] (https://www3.cmbi.umcn.nl/hope) and is depicted in Fig. 2 A-D. We first compared the basic structure of the two amino-acids: leucine, the original wild-type residue, and histidine, the newly introduced mutant, differ in amino acid *size* and *hydrophobicity*-value, and are thus expected to modify the protein properties. First, the mutant residue is bigger than the wild-type residue, and this may lead to bumps; second, the wild-type residue is more hydrophobic than the mutant residue, and thus hydrophobic interactions, either in the core of the protein or on the surface, are expected to be lost. As regards the secondary structure, the wild-type residue is predicted (using the Reprof software) to be located in an α-helix. The variant converts the wild-type residue in one that does not prefer α-helices as secondary structure. With respect to conservation, the wild-type residue is very conserved, but a few other residue types have also been observed at this position (https://www.ncbi.nlm.nih.gov/snp/rs1336224465). The mutant residue is located near a highly conserved position: based on conservation scores, this variant is probably damaging to the protein. Moreover, the mutated residue is located in a domain that is important for binding of other molecules, so that mutation of the residue might disturb this binding function. Thus, although we did not test the functional consequences of the c.1274 T > A variant, there were several indirect evidences of its pathogenic potential, likely through interference with the binding function in the catalytic site.

In conclusion, we found a new missense mutation at site 425 where leucine was replaced by histidine (L425H). Co-segregation of the mutation with the aura phenotype was complete, suggesting a 100% penetrance, although with a mild phenotype. In silico protein prediction softwares indicate that L425H is deleterious by changing the 3D structure of ATPA1A2 at the cytoplasmic loop between the two central transmembrane helices TM4 and TM5 (between site 330 and 700). Further studies taking into account genotype-phenotype correlations, intra-familiar variability, and functional consequences on protein structure and function may help clarify whether this site of mutation is always responsible for relatively milder phenotypes, as in our case, or whether there are other factors (genetic modifiers, epigenetic, environmental?) influencing the clinical expressivity.

## Abbreviations

ACMG: American College of Medical Genetics and Genomics
DTI: diffusion tensor imaging
FHM2: familiar hemiplegic migraine type 2
HM: hemiplegic migraine
MA: migraine with aura
MAF: minor allele frequency
MWA: migraine without aura
NGS: next generation sequencing
PM: pathogenic moderate
PP: pathogenic supporting
SNP: single nucleotide polymorphism
TM: trans-membrane
